# The Impact of Body Mass Index, Residual Beta Cell Function and Estimated Glucose Disposal Rate on the Development of Double Diabetes and Microvascular Complications in Patients With Type 1 Diabetes Mellitus

**DOI:** 10.7759/cureus.48979

**Published:** 2023-11-17

**Authors:** Rameez Raja Bhagadurshah, Subbiah Eagappan, Raghavan Kasthuri Santharam, Sridhar Subbiah

**Affiliations:** 1 Department of Endocrinology and Diabetology, Madurai Medical College and Government Rajaji Hospital, Madurai, IND

**Keywords:** diabetic retinopathy, diabetic nephropathy, body mass index (bmi), india, c-peptide, microvascular complications, estimated glucose disposal rate, double diabetes, type 1 diabetes mellitus (t1dm)

## Abstract

Background

The clinical impact of body mass index (BMI), residual beta cell function and estimated glucose disposal rate (eGDR) in the development of double diabetes (DD) and microvascular complications are largely unknown. We aimed to assess whether BMI, residual beta cell function measured by plasma “C” peptide and insulin resistance measured by eGDR have any impact on the development of DD and microvascular complications in patients with type 1 diabetes mellitus (T1DM).

Methods

It is a cross-sectional observational study involving 113 T1DM patients of more than five years duration who were classified into two groups: normal BMI (18.5-22.9 kg/m^2^) and overweight/obese group (≥ 23kg/m^2^) based on Asian BMI classification. Based on their eGDR values, they were grouped into four categories: ≥ 8, 6-7.99, 4-5.99, and < 4 mg/kg/min. The prevalence of DD based on eGDR values was determined. Their BMI and different eGDR categories were compared with the prevalence of diabetic retinopathy and nephropathy and their odds ratio (OR) was calculated.

Results

The median and interquartile range (IQR) of the eGDR of the overweight/obese group was significantly lower than the normal BMI group (5.3 [3.96-8.15] vs 8.72 [6.50-9.77 mg/kg/min], p < 0.001). The prevalence of DD in the overweight/obese T1DM group and normal BMI group was 75% and 33.3% respectively. The OR of retinopathy and nephropathy in the overweight/obese group was 3.28 (p = 0.007) and 3.01 (p = 0.015) respectively when compared to the normal BMI group. The OR of retinopathy and nephropathy in T1DM patients with eGDR < 4 mg/kg/min was 17.13 (p = 0.001) and 18.5 (p = 0.001) respectively. The lower the eGDR values, the higher the prevalence of retinopathy and nephropathy regardless of HbA1c levels.

Conclusion

As overweight and obesity are increasingly becoming more prevalent in T1DM, the eGDR will better predict the development of DD and microvascular complications irrespective of HbA1c levels. It is more useful as a variable and easily inducted into routine clinical practice. However, residual beta cell function was not useful in predicting the development of microvascular complications.

## Introduction

Type 1 diabetes mellitus (T1DM) is characterized by the autoimmune destruction of pancreatic beta cells leading to severe insulin deficiency [1]. Plasma “C” peptide is a valuable marker of residual beta cell function in diabetes mellitus. Recent studies established that there is some residual insulin secretion persists even after many years of diagnosis in patients with T1DM [2]. Because of the availability of highly sensitive newer assays such as electrochemiluminescence immunoassay (ECLIA), it is possible to quantify the lowest level of plasma “C” peptide. However, the clinical impact of this residual beta cell secretion is largely unknown. Also, how much the minimal level of C-peptide is required for clinical benefit is not known. Only a few studies have been conducted in this regard, which also shows conflicting results. Some studies indicate that even some residual beta cell functions have benefits in preventing the chronic complications of diabetes by aiding in achieving good glycemic control [3,4]. Whereas other studies revealed that this has no clinical benefit in this regard except for reduced incidence of hypoglycemia [5]. Only a few Indian studies are available in these aspects.

Estimated glucose disposal rate (eGDR) is an indirect measure of detecting insulin resistance (IR) and is well validated with hyperinsulinemic-euglycemic clamp in patients with T1DM [6]. Nowadays, obesity and IR are more prevalent among patients with T1DM because of modern lifestyle factors, chronic intensive subcutaneous insulin treatment, and other factors [7,8]. This is also called double diabetes (DD). eGDR is strongly correlated with both microvascular and macrovascular complications, irrespective of HbA1c [9-12]. Hence in this study, we aimed to assess whether residual beta cell function measured by plasma “C” peptide and IR measured by eGDR has any impact on the development of chronic microvascular complications in patients with T1DM of more than five years duration.

## Materials and methods

This cross-sectional study was conducted between June 2022 and August 2023 in the Department of Diabetology of a 3000-bed multispecialty tertiary care hospital (Madurai Medical College and Government Rajaji Hospital, Madurai) in South India. The study was approved by the Institutional Ethics Committee Reg. No. EC/NEW/INST/2022/TN/0059. A total of 113 T1DM patients with a disease duration of more than five years were enrolled in this study. Patients were recruited based on the following inclusion criteria. (i) Confirmed diagnosis of T1DM individuals with classic presentation and on insulin therapy since diagnosis, (ii) diabetes duration more than five years, (iii) age at recruitment more than 18 years old, (iv) BMI ≥ 18.5 kg/m^2^. Pregnant women with T1DM, BMI <18.5 kg/m^2^ were excluded.

Informed written consent was taken from every study subject at the time of their enrolment in the study. Demographic details such as age at diagnosis, duration of diabetes, and family history of type 2 diabetes were collected. Height was measured by a stadiometer in centimetres, weight by a weighing machine in kilograms, and waist and hip circumference by flexible non-elastic measuring tape in centimetres. The BMI is calculated as weight in kilograms divided by height in meters squared. Random capillary blood glucose (CBG) was measured by glucometer irrespective of the last meal. If the CBG is more than 140 mg/dl, a venous blood sample was drawn for plasma glucose and random non-fasting plasma “C” peptide, and their levels were estimated. Random non-fasting plasma “C” peptide was measured by using Roche Cobas e411 - ECLIA Analyzer (Roche Diagnostics GmbH, Mannheim, Germany), by ECLIA technique. HbA1c measured by Bio-Rad D-10 ion (Bio-Rad Laboratories, Hercules, USA) exchange high-performance liquid chromatography (HPLC) method. Spot urine protein creatinine ratio (PCR) measured by immunoturbidimetry method. Fundus examination is done by the ophthalmologist using direct ophthalmoscopy.

As the study population was South Asian, patients with T1DM were categorized into two groups based on Asian BMI classification cut-offs which include the normal BMI group (18.5-22.9 kg/m^2^) and the overweight/obese BMI group (≥ 23kg/m^2^) [13]. Level 3 hypoglycemia is defined as a severe documented hypoglycemic episode with an altered mental state and/or impaired physical functioning that necessitates help from another person for recovery. Nephropathy is defined as the presence of persistent proteinuria and an estimated glomerular filtration rate < 60 ml/min per 1.73m^2^. Persistent proteinuria was defined as when spot urine PCR ≥ 0.5 mg/mg on more than one occasion at least three months apart [14]. Retinopathy is defined as present if the observed fundus abnormalities are more than retinal microaneurysms in at least one eye. Retinopathy was taken into account only when other classical diabetes retinal changes should be present along with retinal microaneurysms.

eGDR* is calculated based on the formula [12]:

eGDR (mg/kg/min) = 19.02-(0.22 x BMI)-(3.26 x HTN)-(0.61 x HbA1c)

BMI - body mass index (kg/m^2^), HTN - hypertension i.e., hypertension was defined as treatment with anti-hypertensive medication, or systolic blood pressure ≥ 140 mmHg, or diastolic blood pressure ≥ 90 mmHg, respectively (yes = 1/no = 0), HbA1c - HbA1c (%)

Based on the previous studies, eGDR was grouped into four categories (<4, 4-5.99, 6-7.99, and ≥8 mg/kg/min). eGDR ≥8 mg/kg/min is considered as no IR whereas eGDR of 4-5.99, 6-7.99, and <4 mg/kg/min is considered as mild, moderate, severe degree of IR. An individual with T1DM with an eGDR value of < 8mg/kg/min was diagnosed as DD [8,12,15,16].

Statistical analyses

All analyses were conducted using Statistical Package for the Social Sciences (IBM SPSS Statistics for Windows, IBM Corp., Version 26.0, Armonk, NY). According to the Shapiro-Wilks test, data was found to be skewed in distribution. Descriptive statistics were computed in median [IQR] or number (%). The Mann-Whitney U test was used to compare and determine the association between different continuous variables. The chi-square test was used to find the association between categorical variables. Spearman rank correlation was used to find the direction of association among chosen variables in the study population. Multiple logistic regression analysis was performed to determine the association of retinopathy and nephropathy with BMI or eGDR categories. The odds ratio (OR) was presented as crude OR and adjusted OR among BMI and eGDR categories. For adjusted OR, age, duration of diabetes, and HbA1c were adjusted for the BMI category whereas age and duration of diabetes were adjusted for the eGDR category. Adjustments for BMI, HbA1c, and the presence of hypertension were not made when evaluating the association between eGDR and different microvascular complications because these variables are part of eGDR calculation similar to previous studies [12,15]. The confidence interval was set at 95%. The results were considered significant with a p-value less than 0.05.

## Results

Baseline characteristics of study cohorts

Among 152 patients identified for the study, 113 patients who met the inclusion criteria were recruited for the primary analysis. In this study, the median age at recruitment was 32 years (IQR: 27-35 years), whereas the median age at diagnosis was 15 years (IQR: 14-18 years) and the median duration of diabetes at the time of the study was 16 years (IQR: 11-20 years). Among 113 patients, 54.9% were males. A family history of type 2 diabetes mellitus (T2DM) was present in 43.4% of the patients. A total of 61.1% of patients were in the normal BMI group whereas 38.9% of patients were in the overweight/obese group. A total of 39.8% of patients were hypertensive at the time of the study. The median non-fasting random “C” peptide was 0.11 ng/ml (IQR: 0.05-0.4 ng/ml) whereas their median HbA1c was 8.8% (IQR: 8.1-9.7%). Median eGDR was 8.1 mg/kg/min (IQR: 5.24-9.36 mg/kg/min). The prevalence of retinopathy and nephropathy in the present study cohorts were 26.5% and 23.9% respectively.

Comparison of baseline characteristics of normal BMI and overweight/obese T1DM patients

The median age of patients and the median age at diagnosis in the normal BMI group were similar when compared to the overweight/obese BMI group (31 vs 32.5 years, p=0.279) and (15 vs 16 years, p = 0.031) respectively. There was no statistically significant difference between these groups with respect to gender, diabetes duration, frequency of diabetic ketoacidosis (DKA) and level 3 hypoglycemia. The overweight/obese BMI group had a significant family history of T2DM when compared to the normal BMI group. Also, hypertension was more frequent in the overweight/obese group than in the normal BMI group. Their non-fasting random “C” peptide levels were similar between normal BMI and overweight/obese group (0.1 vs 0.15 ng/ml, P<0.447). Similarly, no significant difference in the HbA1c between these groups (8.8 vs 9.1%, P = 0.447). The eGDR in the overweight/obese BMI group was significantly lower than the normal BMI group (5.3 vs 8.72 mg/kg/min, P < 0.001). The prevalence of DD based on eGDR in overweight/obese T1DM patients is 75% and 33.3% in normal BMI T1DM patients, which is statistically significant. A comparison of demographics, clinical, and biochemical characteristics of normal BMI and overweight/obese BMI T1DM patients is depicted in Table 1.

**Table 1:** Demographic, clinical and biochemical characteristics of normal BMI and overweight/obese BMI T1DM patients. * Significant p-value, Median [Interquartile range]. DM - Diabetes mellitus, DKA - Diabetic ketoacidosis, BMI - Body mass index, SBP - Systolic blood pressure, DBP - Diastolic blood pressure, HbA1c - Glycosylated haemoglobin, eGDR - Estimated glucose disposal rate, eGFR - Estimated glomerular filtration rate

Variables	Total, n = 113	Normal BMI Group, n = 69	Overweight/Obese BMI Group, n = 44	Normal BMI vs Overweight/Obese BMI, P Value
Age, Years	32 [27-35]	31 [27-35]	32.5 [28-35]	0.279
Male, n (%)	62 [54.9%]	47 [68.1%]	25 [56.8%]	0.223
Age at Diagnosis, Years	15 [14-18]	15 [14-17]	16 [14.5-18]	0.031
Duration of DM, Years	16 [11-20]	15 [10-20]	16 [11-19.5]	0.913
DKA admission in last year, n (%)	19 (16.8%)	11 (15.9%)	8 (18.2%)	0.756
Level 3 Hypoglycemia in last 1 year, n (%)	19 (16.8%)	13 (18.8%)	6 (11.4%)	0.470
Family History of Type 2 DM, n (%)	49 (43.4%)	24 (34.8%)	25 (56.8%)	0.021*
BMI, Kg/m^2^	22.04 [19.29-24.49]	19.53 [18.80-21.70]	24.91 [23.86-26.59]	< 0.001*
SBP, mm Hg	130 [110-140]	120 [110-135]	140 [127.5-150]	<0.001*
DBP, mm Hg	80 [73-90]	80 [70-90]	90 [78-91]	0.015*
Hypertension, n (%)	45 (39.8%)	19 (27.5%)	26 (59.1%)	<0.001*
Residual Random plasma C Peptide, ng/ml	0.11 [0.05-0.4]	0.1 [0.05-0.39]	0.15 [0.07-0.4]	0.447
HbA1c, %	8.8 [8.1-9.7]	8.8 [8.1-9.6]	9.1 [8.15-9.8]	0.251
eGDR, mg/kg/min	8.1 [5.24-9.36]	8.72 [6.5-9.77]	5.3 [3.96-8.15]	<0.001*
eGDR <8 mg/kg/min, n (%) (Double Diabetes)	56 (49.5%)	23 (33.3%)	33 (75%)	<0.001*
Retinopathy, n (%)	30 (26.5%)	12 (17.4%)	18 (40.9%)	0.005*
Nephropathy, n (%)	27 (23.9%)	11 (15.9%)	16 (36.4%)	0.013*
eGFR, ml/min	90 [82-95]	91 [82-95]	90 [81.5-95]	0.388

Microvascular complications such as retinopathy and nephropathy were more prevalent in the overweight/obese group when compared to the normal BMI group. A comparison of baseline characteristics of study cohorts with retinopathy and nephropathy is summarized in Table 2.

**Table 2:** Comparison of clinical and biochemical characteristics with retinopathy and nephropathy in T1DM patients. * Significant p-value, Median [Interquartile range]. T1DM - Type 1 diabetes mellitus, BMI - Body mass index, HbA1c - Glycosylated haemoglobin, SBP - Systolic blood pressure, DBP - Diastolic blood pressure, eGDR - Estimated glucose disposal rate

Variables	T1DM without Retinopathy, n = 83	T1DM with Retinopathy, n = 30	P Value	T1DM without Nephropathy, n = 86	T1DM with Nephropathy, n = 27	P Value
BMI Kg/m^2^	21.1 [18.9-23.7]	23.6 [22.2-26.0]	0.001*	21.1 [19.0-23.7]	23.7 [22.2-25.2]	0.001*
C Peptide, ng/ml	0.11 [0.04-0.3]	0.15 [0.07-0.4]	0.161	0.10 [0.05-0.4]	0.10 [0.07-0.4]	0.170
HbA1c %	8.5 [7.95-9.3]	9.55 [9.1-9.8]	0.001*	8.6 [7.9-9.3]	9.7 [9.25-10.4]	0.001*
SBP mm Hg	120 [110-140]	142 [131-146]	0.001*	120 [110-140]	142 [130-143]	0.001*
DBP mm Hg	80 [70-90]	90 [80-90]	0.015*	80 [70-90]	90 [80-90]	0.015*
Hypertension, n (%)	18 (21.7%)	27 (90.0%)	0.001*	21 (24.4%)	24 (88.9%)	0.001*
eGDR, mg/kg/min	8.62 [6.89-9.69]	4.96 [3.78-5.35]	0.001*	8.58 [6.52-9.68]	4.92 [3.82-5.31]	0.001*

eGDR and microvascular complications

**Figure 1:**
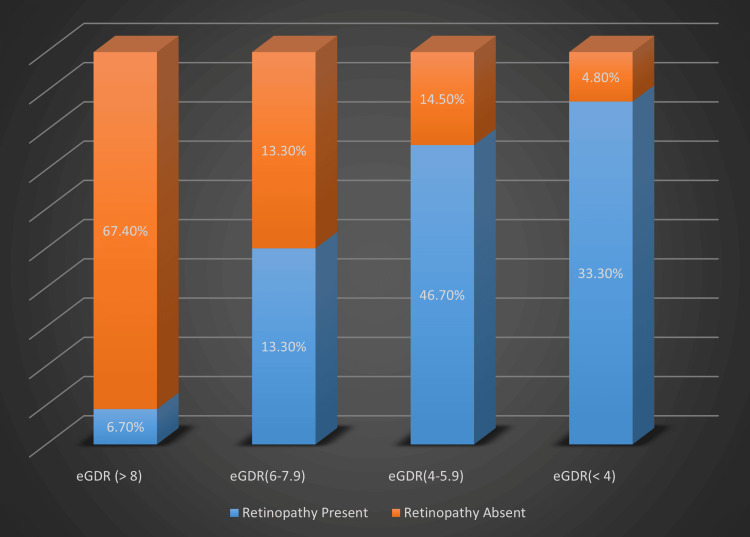
Relationship between different eGDR categories and prevalence of retinopathy in T1DM patients. eGDR - Estimated glucose disposal rate (mg/kg/min). Study participants were grouped into four different eGDR categories as shown in X-axis. The percentage of T1DM patients with retinopathy and without retinopathy in each eGDR group is shown in Y-axis.

**Figure 2:**
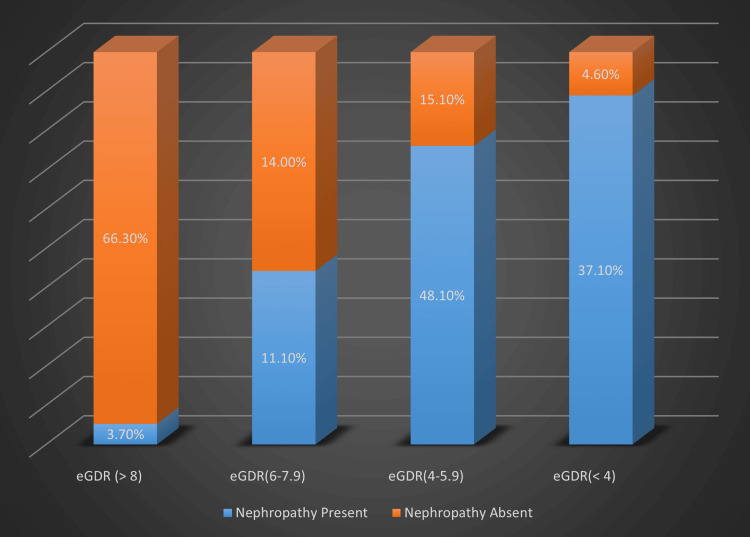
Relationship between different eGDR categories and prevalence nephropathy in T1DM patients. eGDR - Estimated glucose disposal rate (mg/kg/min). Study cohorts were grouped into four different eGDR categories as shown in X-axis. The percentage of T1DM patients with nephropathy and without nephropathy in each eGDR group is shown in Y-axis.

The eGDR ≥ 8 mg/kg/min is associated with a lower risk of microvascular complications whereas eGDR < 8 mg/kg/min is associated with a higher risk of these complications irrespective of HbA1c levels. eGDR ≥ 8 had the lowest risk of developing microvascular complications whereas eGDR < 4 and 4-5.99 had the highest risk of developing these microvascular complications. Four different categories of eGDR compared with the prevalence of retinopathy and nephropathy among T1DM patients are depicted in Table 3, Figure 1 and Figure 2.

**Table 3:** Comparison of estimated glucose disposal rate (eGDR) with retinopathy and nephropathy in T1DM patients. * Significant p value. T1DM - Type 1 diabetes mellitus, eGDR - Estimated glucose disposal rate

eGDR, mg/kg/min	T1DM without Retinopathy, n = 83	T1DM with Retinopathy, n = 30	P Value	T1DM without Nephropathy, n = 86	T1DM with Nephropathy, n = 27	P Value
< 4	4 (4.8%)	10 (33.3%)	0.001*	4 (4.6%)	10 (37.1%)	0.001*
4-5.99	12 (14.5%)	14 (46.7%)		13 (15.1%)	13 (48.1%)	
6-7.99	11 (13.3%)	4 (13.3%)		12 (14.0%)	3 (11.1%)	
> 8	56 (67.4%)	2 (6.7%)		57 (66.3%)	1 (3.7%)	

Correlation of clinical and biochemical characteristics of study cohorts with retinopathy and nephropathy

eGDR, BMI and hypertension had a significant inverse correlation with the presence of retinopathy and nephropathy. Whereas age at diagnosis and residual plasma C peptide levels had no significant correlation with these microvascular complications. Although the duration of diabetes had a strong positive correlation with retinopathy, it had a weak correlation with nephropathy. In contrast, HbA1c is strongly correlated with nephropathy but it is weakly correlated with retinopathy. Data correlation of the residual beta cell function, duration of diabetes, eGDR, BMI, HbA1c, hypertension with retinopathy and nephropathy in T1DM patients is provided in Table 4.

**Table 4:** Correlation of residual beta cell function, duration of diabetes, estimated glucose disposal rate (eGDR), BMI, HbA1c, hypertension with retinopathy and nephropathy in T1DM patients. * Significant p-value. eGDR - Estimated glucose disposal rate, BMI - Body mass index, HbA1c - Glycosylated haemoglobin Residual beta cell function was measured by residual random plasma "C" peptide in ng/ml.

Parameters	Retinopathy	P value	Nephropathy	P value
Residual plasma “C” peptide (ng/ml)	0.096	0.308	0.090	0.342
Duration of diabetes (years)	0.294	0.001*	0.166	0.078
eGDR (mg/kg/min)	-0.613	<0.001*	-0.612	<0.001*
BMI (Kg/m^2^)	0.356	0.001*	0.273	0.003*
HbA1c (%)	0.171	0.069	0.339	<0.001*
Hypertension	0.610	<0.001*	0.561	<0.001*

Impact of BMI, eGDR on the prevalence of retinopathy and nephropathy in T1DM patients

**Figure 3:**
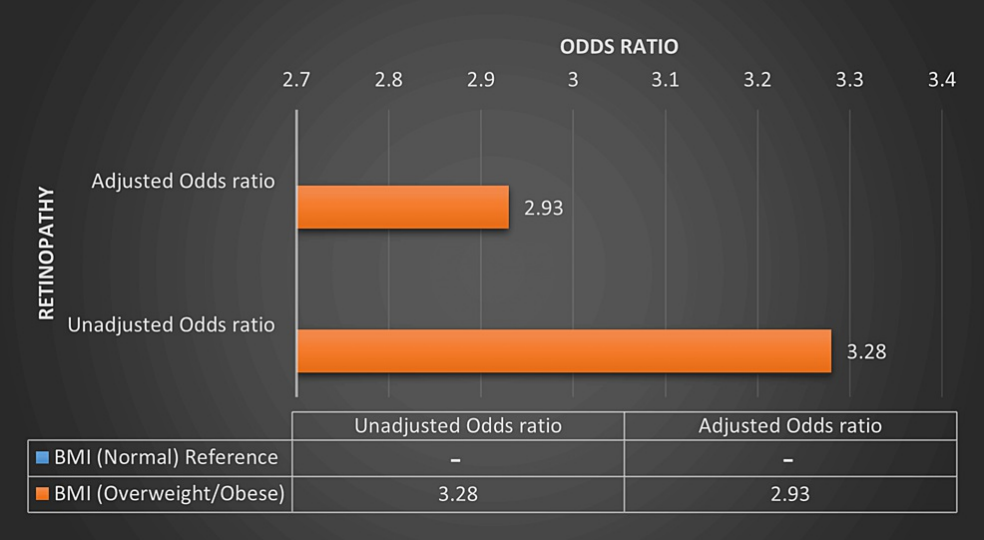
Multiple logistic regression analysis showing the odds ratio of retinopathy based on BMI categories in T1DM patients. BMI - Body mass index

**Figure 4:**
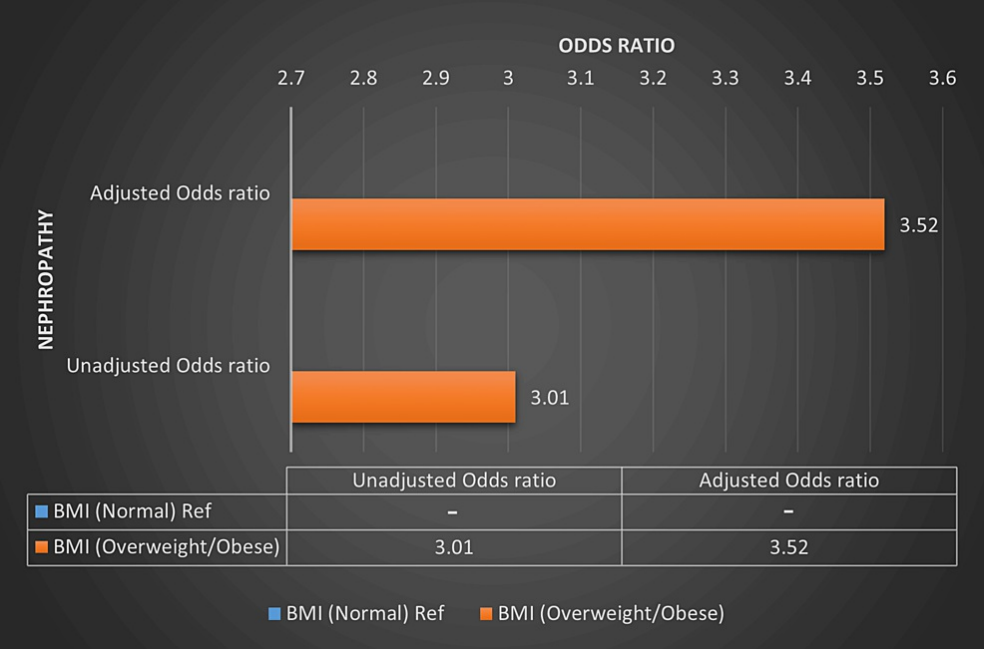
Multiple logistic regression analysis showing the odds ratio of nephropathy based on BMI categories in T1DM patients. BMI - Body mass index

The OR of retinopathy and nephropathy in the overweight/obese group when compared to the normal BMI group is 3.28 (p = 0.007) and 3.01 (p = 0.015) in an unadjusted model, where it is 2.93 (p = 0.045) and 3.52 (p = 0.029) in an adjusted model (adjusted for age, gender, HbA1c and diabetes duration) respectively. For BMI, a significantly higher OR of developing retinopathy and nephropathy was seen in the overweight/obese BMI group when compared to the normal BMI group in both unadjusted and adjusted models (adjusted for age, HbA1c and diabetes duration). Multiple logistic regression analysis showing the OR of retinopathy and nephropathy based on BMI categories is summarized in Table 5, Figure 3 and Figure 4.

**Table 5:** Multiple logistic regression analysis showing the odds ratio of retinopathy and nephropathy based on BMI categories in T1DM patients. ** Adjusting variables were age, HbA1C, duration of diabetes; R2=0.60; The model explained 60% (Nagelkerke R2) of the variance in microvascular complication with Percentage accuracy in classification (PAC) at 77%. * Significant p value. BMI - Body mass index

Parameters	Unadjusted Odds Ratio	P Value	Adjusted Odds Ratio**	P Value
Retinopathy				
BMI (Normal)	Reference	-	Reference	-
BMI (Overweight/Obese)	3.28	0.007*	2.93	0.045*
Nephropathy				
BMI (Normal)	Reference	-	Reference	-
BMI (Overweight/Obese)	3.01	0.015*	3.52	0.029*

**Figure 5:**
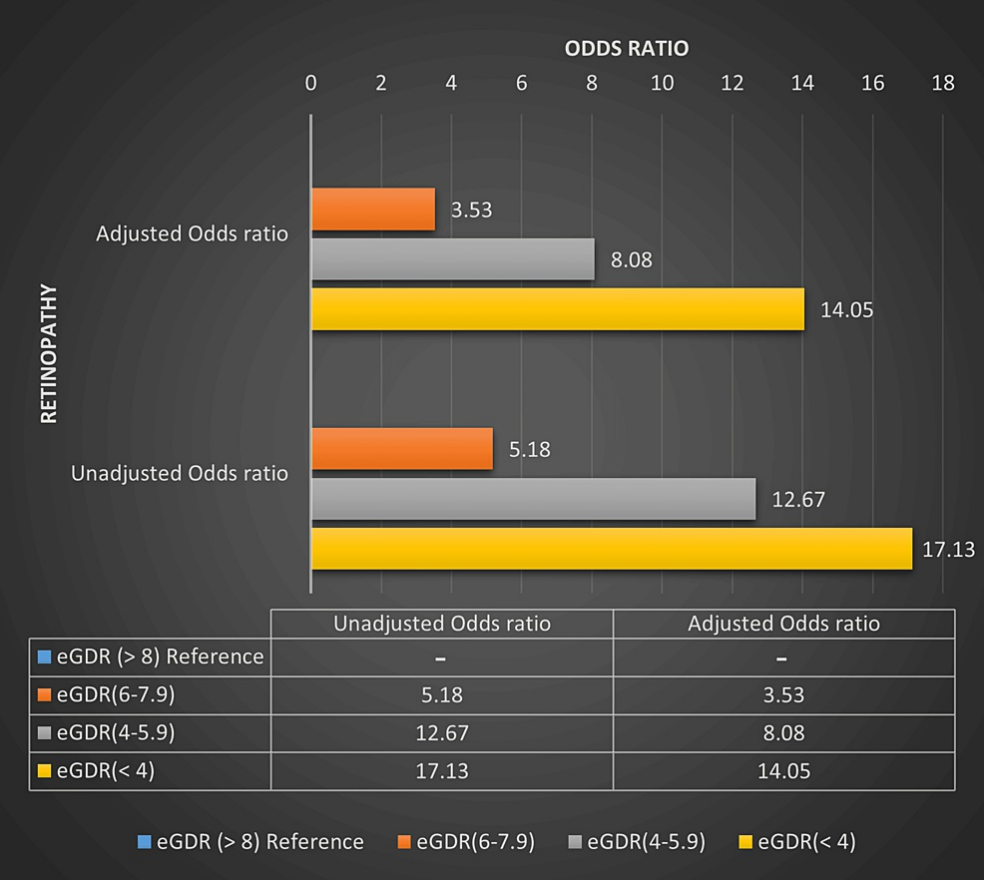
Multiple logistic regression analysis showing the odds ratio of retinopathy based on different eGDR categories in T1DM patients. eGDR - Estimated glucose disposal rate

**Figure 6:**
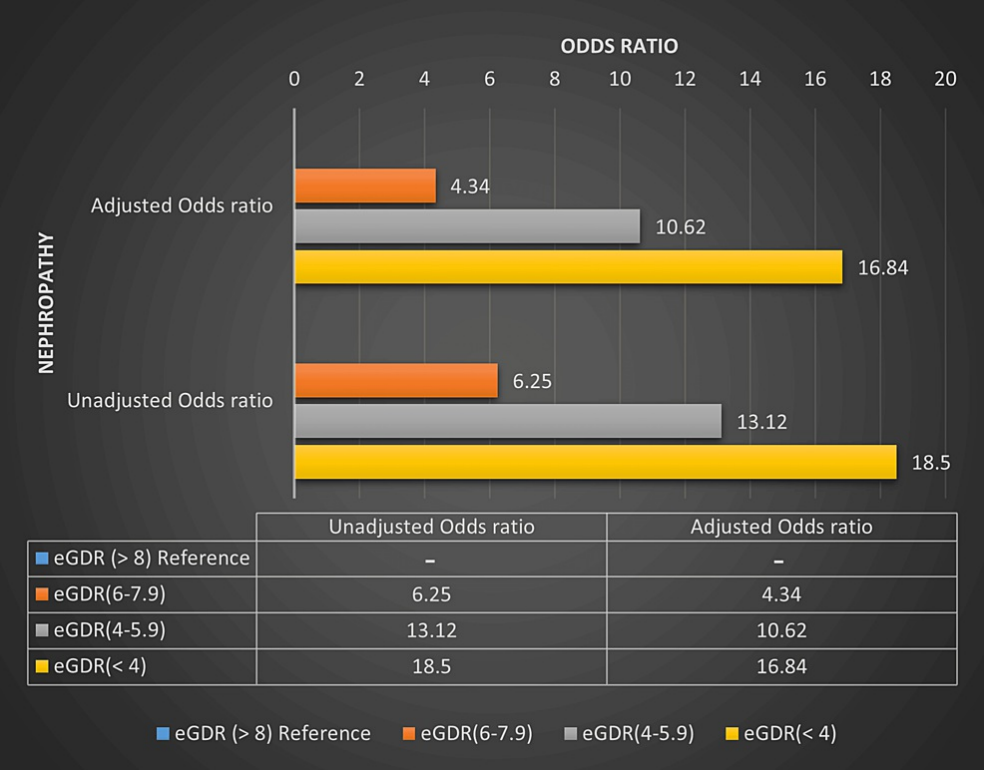
Multiple logistic regression analysis showing the odds ratio of nephropathy based on different eGDR categories in T1DM patients. eGDR - Estimated glucose disposal rate

The OR of retinopathy and nephropathy in T1DM patients with eGDR < 4 mg/kg/min is 17.13 (p = 0.001) and 18.5 (p = 0.001) in an unadjusted model whereas it was 14.05 (p=0.001) and 16.84 (p=0.001) in an adjusted model (adjusted for age and duration of diabetes) respectively. Similarly, there is a statistically significantly higher OR of retinopathy and nephropathy with other eGDR categories (6-7.99, 4-5.99 mg/kg/min) also noted. Multiple logistic regression analysis showing the OR of retinopathy and nephropathy based on different eGDR categories is summarized in Table 6, Figure 5 and Figure 6.

**Table 6:** Multiple logistic regression analysis showing the odds ratio of retinopathy and nephropathy based on different eGDR categories in T1DM patients. ** Adjusting variables were age, duration of diabetes; R2=0.62; The model explained 62% (Nagelkerke R2) of the variance in microvascular complication with percentage accuracy in classification (PAC) at 81.4%. * Significant p-value eGDR - Estimated glucose disposal rate

Parameters	Unadjusted Odds Ratio	P value	Adjusted Odds Ratio**	P value
Retinopathy				
eGDR (> 8)	Reference	-	Reference	-
eGDR (6-7.9)	5.18	0.012*	3.53	0.036*
eGDR (4-5.9)	12.67	0.001*	8.08	0.001*
eGDR (< 4)	17.13	0.001*	14.05	0.001*
Nephropathy				
eGDR (> 8)	Reference	-	Reference	-
eGDR (6-7.9)	6.25	0.027*	4.34	0.047*
eGDR (4-5.9)	13.12	0.001*	10.62	0.001*
eGDR (< 4)	18.5	0.001*	16.84	0.001*

Longer duration of diabetes, higher BMI, higher HbA1c, presence of hypertension, and lower eGDR increased the risk and predicted the development of retinopathy and nephropathy in T1DM patients whereas age at diagnosis and residual plasma “C” peptide secretion were not useful in predicting the risk of development of these microvascular complications.

## Discussion

This study highlights the increased prevalence of overweight and obesity in adults with T1DM. eGDR, a surrogate marker of IR, predicted the development of microvascular complications in these patients. T1DM patients with eGDR ≥ 8 mg/kg/min had a lower risk of development of retinopathy and nephropathy whereas eGDR < 8 mg/kg/min had an increased risk of developing these microvascular complications.

Overweight and obese T1DM patients are usually associated with lower eGDR i.e., <8 mg/kg/min, and had an increased prevalence of retinopathy and nephropathy when compared to that of normal BMI T1DM patients.

The prevalence of overweight/obesity in this study was 38.9% which is lower when compared to other studies done in England by Cantley et al. [17]. However, this prevalence was close to the prevalence of overweight and obesity in the general population of South India [18]. This implied that patients with T1DM had the same risk of developing overweight and obesity as the general population. This will pave the way for more research needed to understand the risk factors of developing overweight and obesity and its complications in T1DM patients. Thereby, treatment strategies to prevent overweight and obesity in these individuals can be devised.

The age at onset of T1DM was similar between normal BMI and overweight/obese groups. This indicated that age at onset of T1DM had no impact on the development of overweight/obesity in subsequent years of life. However, there is a strong association between the duration of T1DM and overweight/obesity development. Many factors contribute to overweight/obesity in T1DM which include longer duration of insulin treatment which induces weight gain and thereby increases peripheral IR, and sedentary lifestyles with contribution from increasing age [19]. This creates a vicious cycle that longer duration insulin treatment increases weight gain and IR which in turn increases insulin requirement for controlling hyperglycemia which in turn again leads to weight gain [20]. The strong association between insulin treatment and weight gain leads to difficulty in managing diabetes in these patients. The prevalence of DKA, level 3 hypoglycemia was similar between normal weight and overweight/obese T1DM patients. This suggested that both these two groups are equally susceptible to DKA and hypoglycemia as residual C peptide secretion was very low and similar in these patients.

Interestingly, a family history of T2DM was more prevalent in overweight/obese T1DM patients when compared to normal BMI T1DM patients. Similarly, a family history of T2DM was common in other studies conducted on T1DM patients. This includes one study done by Teupe and Bergis, who identified there was a strong family history of T2DM in 15% of T1DM patients who had higher BMI [21]. A larger Finnish study done in 1860 T1DM patients identified that 33% of them had a strong family history of T2DM, who again had higher BMI [22]. Diabetes Control and Complications Trial (DCCT) has shown that family history of T2DM in patients with T1DM had greater development of obesity, require higher insulin dose, and abnormal lipid profile [20]. This was explained by the fact that T1DM has a genetic predisposition to IR especially in those with a strong family history of T2DM and are prone to overweight/obesity, higher body fat composition, and IR [23].

The prevalence of hypertension was 59% among T1DM patients with overweight/obesity whereas 27.5% in normal-weight T1DM patients. Long-duration of diabetes, hyperglycemia, overweight/obesity, and associated IR are the risk factors for the development of hypertension [20,24,25]. Among these risk factors, obesity plays an important role in inducing IR, activation of the sympathetic nervous system, increased renin-angiotensin-aldosterone system activity, and endothelial dysfunction [26,27]. Hypertension is considered to be an important risk for the development of microvascular and macrovascular complications.

Glycemic control was assessed by HbA1c in patients with T1DM. Although higher HbA1c was seen in both normal-weight and overweight/obesity T1DM individuals, there is no statistical difference in HbA1c between them. However, median HbA1c was 0.3% higher in the overweight/obese group than the normal BMI group. This showed the increasing trend in HbA1c in overweight/obese patients and a larger sample size would be needed to demonstrate this trend in a significant manner [12,20]. In a US-based multicentre study, HbA1c was higher in obese adults when compared with normal BMI adults [28].

The eGDR is a surrogate marker of IR. As per various study data available, eGDR less than 8 mg/kg/min is associated with IR whereas eGDR more than 8 mg/kg/min is associated with no IR [8,12]. As the eGDR level decreases, IR proportionately increases in the present study. Median eGDR is 8.72 mg/kg/min in normal BMI patients compared to 5.3 mg/kg/min in overweight/obese patients in this study. This implied that overweight/obese T1DM patients had a greater degree of IR compared to virtually no IR in normal BMI T1DM patients. This is similar to other studies done by Helliwell, Rebecca et al. and Cantley et al. [12,17].

The prevalence of microvascular complications such as retinopathy, and nephropathy was higher in overweight obese T1DM patients than that of normal weight T1DM patients. This is supported by the other studies that showed a similar higher prevalence of these microvascular complications in overweight/obese T1DM patients [12,17]. Many factors contribute to the development of microvascular complications in overweight/obese patients which include obesity-associated IR, hypertension, higher HbA1c, and longer duration of diabetes.

In this present study, longer duration of diabetes, higher BMI, higher HbA1c, presence of hypertension, and lower eGDR were the predictors of microvascular complications. Among these parameters, eGDR is suggested to be the strongest predictor of the development of these microvascular complications, as it includes BMI, HbA1c, presence of hypertension as its components. Whereas residual C peptide secretion (a marker of residual beta cell function) did not predict the presence of microvascular complications in these T1DM patients which is similar to another study done by Marren et al. [5].

In our study, overweight/obese T1DM patients were hypertensive and had higher IR, lower eGDR (<8 mg/kg/min), and a higher prevalence of microvascular complications irrespective of HbA1c levels. Interventions such as low carbohydrate diets, regular physical activity, and use of drugs such as metformin, GLP-1 agonists, and amylin analogs in DD have to be explored with further studies [29].

This observational study has several major strengths. This includes a good mix of rural and urban individuals in a single-centre study. Limitations of the study include that the variations in complications that are triggered by different factors may not be identifiable due to the constraints of our relatively small sample size. Another restricting factor was the selection criteria used in this study, which led to an unequal distribution of participants in the study groups, as both the cause and effect were measured at a single point in time. Since this study is cross-sectional, it is challenging to establish a clear understanding of temporality and biological plausibility.

In light of these limitations, it is essential to consider the potential implications for future research. Consequently, we recommend that future research endeavours prioritize conducting more comprehensive analytical studies with larger sample sizes to address these limitations and provide a more robust understanding of the subject matter.

## Conclusions

In the current world, overweight and obesity are increasingly more prevalent in patients with type 1 diabetes and lead to the development of IR. IR measured by eGDR acts as the better predictor of the development of DD and microvascular complications as it incorporates BMI, HbA1c, and hypertension as a variable within it. It is not only useful as a well-validated tool to stratify the risk of microvascular complications but also useful to monitor the response to change in therapy in patients with type 1 diabetes. Therefore, it can be more useful as a variable in risk stratification in type 1 diabetes and easily inducted into routine clinical practice. However, residual beta cell function measured by plasma “C” peptide is not well correlated with the prevalence of microvascular complications. Hence, it cannot be used as the predictor of the development of microvascular complications in patients with type 1 diabetes.

## References

[1] Eisenbarth GS (1986). Type I diabetes mellitus. A chronic autoimmune disease. N Engl J Med.

[2] Oram RA, McDonald TJ, Shields BM (2015). Most people with long-duration type 1 diabetes in a large population-based study are insulin microsecretors. Diabetes Care.

[3] Rickels MR, Evans-Molina C, Bahnson HT (2020). High residual C-peptide likely contributes to glycemic control in type 1 diabetes. J Clin Invest.

[4] Jeyam A, Colhoun H, McGurnaghan S (2021). Clinical impact of residual C-peptide secretion in type 1 diabetes on glycemia and microvascular complications. Diabetes Care.

[5] Marren SM, Hammersley S, McDonald TJ (2019). Persistent C-peptide is associated with reduced hypoglycaemia but not HbA(1c) in adults with longstanding type 1 diabetes: evidence for lack of intensive treatment in UK clinical practice?. Diabet Med.

[6] Williams KV, Erbey JR, Becker D, Arslanian S, Orchard TJ (2000). Can clinical factors estimate insulin resistance in type 1 diabetes?. Diabetes.

[7] Hother-Nielsen O, Schmitz O, Bak J, Beck-Nielsen H (1987). Enhanced hepatic insulin sensitivity, but peripheral insulin resistance in patients with type 1 (insulin-dependent) diabetes. Diabetologia.

[8] Kietsiriroje N, Pearson S, Campbell M, Ariëns RA, Ajjan RA (2019). Double diabetes: a distinct high-risk group?. Diabetes Obes Metab.

[9] Donga E, Dekkers OM, Corssmit EP, Romijn JA (2015). Insulin resistance in patients with type 1 diabetes assessed by glucose clamp studies: systematic review and meta-analysis. Eur J Endocrinol.

[10] Girgis CM, Scalley BD, Park KE (2012). Utility of the estimated glucose disposal rate as a marker of microvascular complications in young adults with type 1 diabetes. Diabetes Res Clin Pract.

[11] O'Mahoney LL, Kietsiriroje N, Pearson S, West DJ, Holmes M, Ajjan RA, Campbell MD (2021). Estimated glucose disposal rate as a candidate biomarker for thrombotic biomarkers in T1D: a pooled analysis. J Endocrinol Invest.

[12] Helliwell R, Warnes H, Kietsiriroje N, Campbell M, Birch R, Pearson SM, Ajjan RA (2021). Body mass index, estimated glucose disposal rate and vascular complications in type 1 diabetes: beyond glycated haemoglobin. Diabet Med.

[13] WHO Expert Consultation (2004). Appropriate body-mass index for Asian populations and its implications for policy and intervention strategies. Lancet.

[14] Kamińska J, Dymicka-Piekarska V, Tomaszewska J, Matowicka-Karna J, Koper-Lenkiewicz OM (2020). Diagnostic utility of protein to creatinine ratio (P/C ratio) in spot urine sample within routine clinical practice. Crit Rev Clin Lab Sci.

[15] Nyström T, Holzmann MJ, Eliasson B, Svensson AM, Sartipy U (2018). Estimated glucose disposal rate predicts mortality in adults with type 1 diabetes. Diabetes Obes Metab.

[16] Pathak V, Mishra I, Baliarsinha AK, Choudhury AK (2022). Prevalence of insulin resistance in type 1 diabetes mellitus and its correlation with metabolic parameters: the double trouble. Eurasian J Med.

[17] Cantley NW, Lonnen K, Kyrou I, Tahrani AA, Kahal H (2021). The association between overweight/obesity and double diabetes in adults with type 1 diabetes; a cross-sectional study. BMC Endocr Disord.

[18] Pradeepa R, Anjana RM, Joshi SR (2015). Prevalence of generalized &amp; abdominal obesity in urban &amp; rural India - the ICMR-INDIAB Study (Phase-I) [ICMR- NDIAB-3]. Indian J Med Res.

[19] Russell-Jones D, Khan R (2007). Insulin-associated weight gain in diabetes - causes, effects and coping strategies. Diabetes Obes Metab.

[20] Purnell JQ, Hokanson JE, Marcovina SM, Steffes MW, Cleary PA, Brunzell JD (1998). Effect of excessive weight gain with intensive therapy of type 1 diabetes on lipid levels and blood pressure: results from the DCCT. Diabetes Control and Complications Trial. JAMA.

[21] Teupe B, Bergis K (1991). Epidemiological evidence for ‘double diabetes’. Lancet.

[22] Thorn LM, Forsblom C, Wadén J (2009). Effect of parental type 2 diabetes on offspring with type 1 diabetes. Diabetes Care.

[23] Arslanian SA, Bacha F, Saad R, Gungor N (2005). Family history of type 2 diabetes is associated with decreased insulin sensitivity and an impaired balance between insulin sensitivity and insulin secretion in white youth. Diabetes Care.

[24] de Boer IH, Kestenbaum B, Rue TC (2008). Insulin therapy, hyperglycemia, and hypertension in type 1 diabetes mellitus. Arch Intern Med.

[25] Pinhas-Hamiel O, Levek-Motola N, Kaidar K (2015). Prevalence of overweight, obesity and metabolic syndrome components in children, adolescents and young adults with type 1 diabetes mellitus. Diabetes Metab Res Rev.

[26] Polsky S, Ellis SL (2015). Obesity, insulin resistance, and type 1 diabetes mellitus. Curr Opin Endocrinol Diabetes Obes.

[27] Landsberg L, Aronne LJ, Beilin LJ, Burke V, Igel LI, Lloyd-Jones D, Sowers J (2013). Obesity-related hypertension: pathogenesis, cardiovascular risk, and treatment: a position paper of The Obesity Society and the American Society of Hypertension. J Clin Hypertens (Greenwich).

[28] Noor N, Akturk HK, Desimone M (2022). 961-P: The effect of obesity on HbA1c among adults with type 1 diabetes: a U.S. based multicenter study. Diabetes.

[29] Cai X, Lin C, Yang W, Nie L, Ji L (2021). Non-insulin antidiabetes treatment in type 1 diabetes mellitus: a systematic review and meta-analysis. Diabetes Metab J.

